# P-1340. Activity of Delafloxacin and Comparator Agents Against Bacterial Isolates From Patients with Diabetic Foot Infection in the United States and Europe (2017–2023)

**DOI:** 10.1093/ofid/ofaf695.1528

**Published:** 2026-01-11

**Authors:** Michael D Huband, Marisa Winkler, Kelley A Fedler, Mariana Castanheira

**Affiliations:** Element, North Liberty, IA; Element Materials Technology/Jones Microbiology Institute, North Liberty, Iowa; Element, North Liberty, IA; Element, North Liberty, IA

## Abstract

**Background:**

Delafloxacin (DLX) is a fluoroquinolone antibacterial with oral and intravenous treatment options approved in ≥50 countries for treatment of acute bacterial skin and skin structure infection or community-acquired pneumonia in adults. Indicated organisms in the United States (US) include: staphylococci (*S. aureus* [SA] both MSSA and MRSA, *S. haemolyticus* and *S. lugdunensis*), streptococci (*S. pyogenes*, *S. agalactiae* and *S. anginosus* group), *Enterococcus faecalis* (EF), Enterobacterales (*Escherichia coli*, *Klebsiella pneumoniae* and *Enterobacter cloacae* species complex) and *Pseudomonas aeruginosa* (PSA).The *in vitro* activity of DLX, ciprofloxacin (CIP), levofloxacin (LEV), moxifloxacin (MOX), minocycline and meropenem-vaborbactam were determined against bacterial isolates from patients with diabetic foot infection (DFI) in the US and Europe (EUR).

**Figure:**
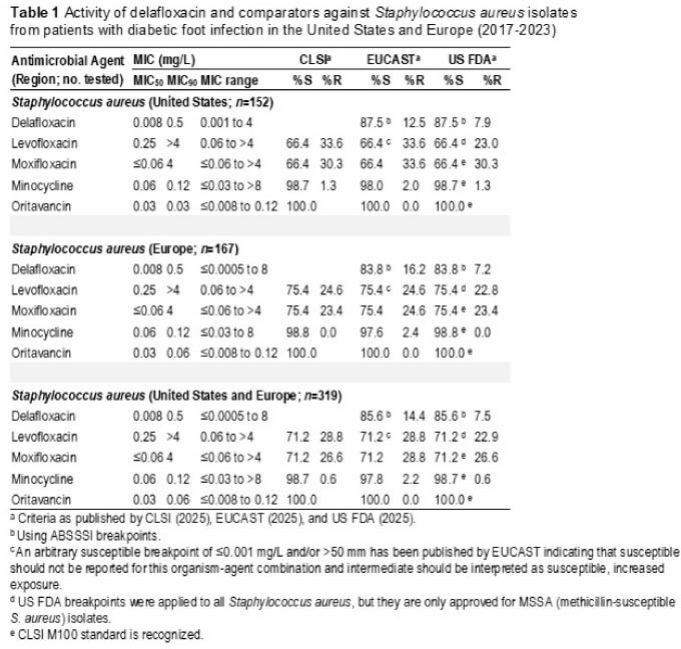

**Figure:**
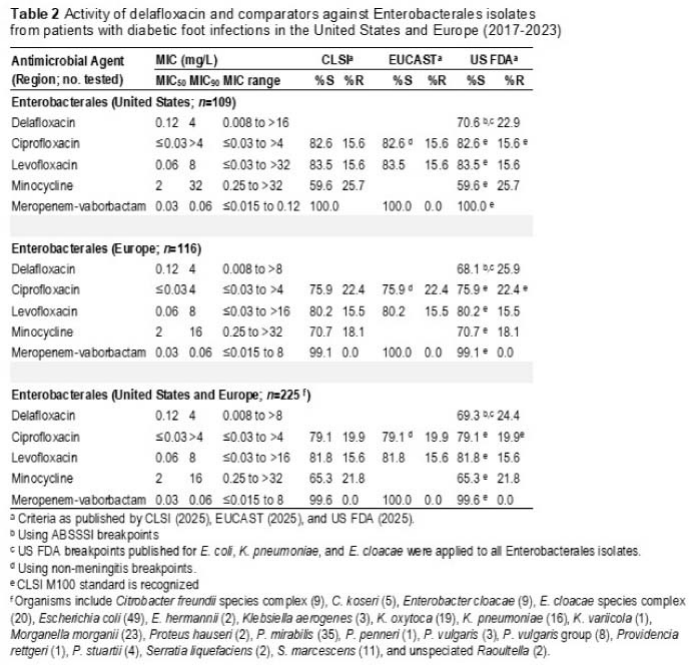

**Methods:**

Isolates from patients with DFI were collected in the SENTRY Surveillance Program (2017–2023) from sites in the US and EUR. Broth microdilution MIC testing was conducted according to CLSI methods. CLSI, EUCAST, and FDA breakpoint criteria (2025) were applied. Isolate identifications were determined at the collection site and confirmed using MALDI-TOF MS (as needed).

**Figure:**
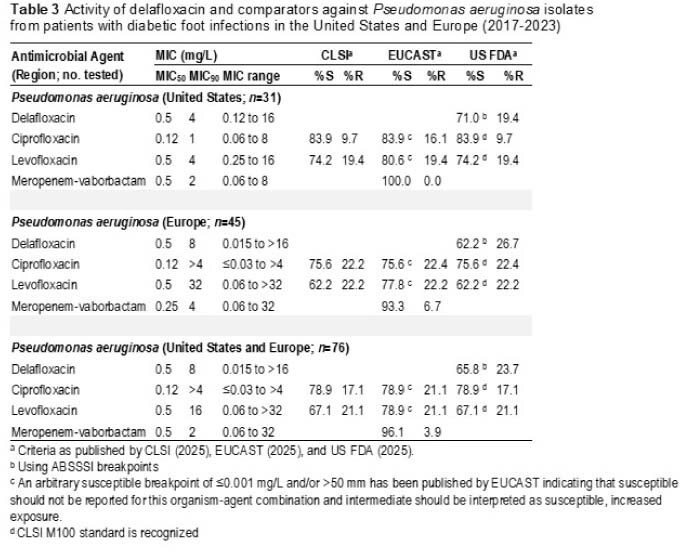

**Results:**

The most common DFI pathogens were SA (35.7%), Enterobacterales (33.4%), PSA (9.8%), streptococci (9.0%) and EF (4.7%). Overall, 87.5% (FDA) of SA isolates from patients with DFI in the US were susceptible to DLX (including 96.7% of MSSA and 73.3% of MRSA) as were 83.8% of SA isolates from EUR (including 96.9% of MSSA and 41.0% of MRSA) (Table 1). Corresponding LEV and MOX susceptibilities for SA isolates from the US were 66.4% (FDA). DLX, CIP and LEV were active (FDA) against 70.6%, 82.6% and 83.5% of Enterobacterales isolates from the US and 68.1%, 75.9% and 80.2% (FDA) of Enterobacterales isolates from EUR, respectively (Table 2). DLX, CIP and LEV were active (FDA) against 71.0%, 83.9% and 74.2% of PSA isolates from the US and 62.2%, 75.6% and 62.2% (FDA) of PSA isolates from EUR, respectively (Table 3).

**Conclusion:**

DLX may provide a useful treatment option (alone or in combination) for patients with DFI including those where a broad-spectrum agent is desired. Additional clinical studies against DFI are needed.

**Disclosures:**

Michael D. Huband, BS, Melinta Therapeutics: Advisor/Consultant|Melinta Therapeutics: Grant/Research Support Marisa Winkler, MD, PhD, Basilea: Advisor/Consultant|Basilea: Grant/Research Support|GSK: Advisor/Consultant|GSK: Grant/Research Support|Melinta Therapeutics: Advisor/Consultant|Melinta Therapeutics: Grant/Research Support|Mundipharma: Advisor/Consultant|Mundipharma: Grant/Research Support|Pfizer: Advisor/Consultant|Pfizer: Grant/Research Support|Pulmocide: Advisor/Consultant|Pulmocide: Grant/Research Support Kelley A. Fedler, BS, Melinta Therapeutics: Grant/Research Support Mariana Castanheira, PhD, Melinta Therapeutics: Advisor/Consultant|Melinta Therapeutics: Grant/Research Support

